# Recombinant VP1 regulates migration of cancer cells and macrophages differentially via let-7a and MMP-9

**DOI:** 10.1186/2051-1426-3-S2-P406

**Published:** 2015-11-04

**Authors:** Shu-Mei Liang, Chi-Ming Liang, Chiao-Chun Liao

**Affiliations:** 1 Agricultural Biotechnology Research Center, Taipei, Taiwan; 2 Genomics Research Center, Academia Sinica, Taipei, Taiwan

## 

Recombinant capsid protein VP1 (rVP1) of foot-and-mouth disease virus is a protein exhibiting potent cytotoxicity to a variety of cancer cells. We have recently found that rVP1 is capable of decreasing cancer cell motility but increasing macrophages motility. The underlying molecular mechanism for such paradoxical effects of rVP1 on cancer and immune cells are still largely unclear. Here, we showed that the miRNA expression patterns in ovarian cancer SKOV-3 cells and RAW264.7 macrophage cells were distinctively different. rVP1 upregulated more let-7a in SKOV-3 cells than in macrophages. Examination of the trajectories and migration velocity of the cells with time-lapse microscopy showed that let-7a was effective in inhibiting SKOV-3 cells migration as compared to scramble control. Pre-treatment with antisense sequence of let-7a blocked the inhibitory effects of not only let-7a but also rVP1 on the migration of SKOV-3 cells. In comparison, RAW264.7 cells which contain more constitutive let-7a than SKOV-3 was not as sensitive as SKOV-3 to the inhibitory effect of synthetic let-7a. RAW264.7 cells were selectively induced by rVP1 to secrete MMP-9 to facilitate its migration, whereas SKOV-3 cells were not. These results thus demonstrated that rVP1 might differentially regulate cell motility by inducing more let-7a or MMP-9 in cancer and immune cells.

